# PSMA-Targeted Radiopharmaceuticals in Prostate Cancer: Current Data and New Trials

**DOI:** 10.1093/oncolo/oyac279

**Published:** 2023-02-18

**Authors:** Brian Ramnaraign, Oliver Sartor

**Affiliations:** Department of Medicine, University of Florida College of Medicine, Gainesville, FL, USA; Department of Medicine, Tulane University School of Medicine, New Orleans, LA, USA

**Keywords:** prostate cancer, PSMA, 177Lutetium, radiopharmaceuticals, VISION trial

## Abstract

Radiopharmaceuticals have been utilized for men with advanced prostate cancer for decades. Older agents, seldom used today, provided palliation for bone metastatic pain. In 2013, the alpha emitter radium-223 provided a catalyst for the field by prolonging survival in men with metastatic castrate-resistant prostate cancer (mCRPC). Recently radioisotopic therapies have gained further interest with the development and FDA approval of 177 lutetium (177Lu)-PSMA-617 (also known as lutetium Lu-177 vipivotide tetraxetan). This agent targets the prostate-specific membrane antigen (PSMA) expressed on the cell surface of prostate cancer cells with a beta-emitting isotope (177Lu). This clinical review summarizes key data reported from 177Lu-PSMA-617 clinical trials, including data from the phase III VISION trial which were pivotal for regulatory approval in heavily pretreated PSMA-PET-positive patients with mCRPC. The current field of radiopharmaceuticals is in a rapid state of flux. Additional phase III trials are now ongoing in patients with mCRPC and in patients with metastatic castrate-sensitive prostate cancer. The results from these potential practice-changing trials are highly anticipated. Earlier phase trials (I/II) are in progress examining combination therapies, radiolabeled monoclonal antibodies, and novel compounds. Studies of PSMA-targeted therapies using both beta emitters such as 177Lu and novel alpha emitters such 225 actinium are in progress. During the next decade, radiopharmaceuticals will likely play a central role in the management of patients with advanced prostate cancer.

## Teaser

A review on the current landscape of radiopharmaceutical therapy in prostate cancer as well as future directions.

**Implications for Practice**

This review summarizes the current and future role of radiopharmaceutical therapies in the management of patients with advanced prostate cancer.

## Background

### Demographics of Prostate Cancer

Prostate cancer is the most common nonskin cancer among men with the American Cancer Society estimating that in 2022, there will be 268 490 new cases of prostate cancer and 34 500 prostate cancer–related deaths.^1^

The incidence of distant prostate cancer is increasing with metastatic disease accounting for 8% of all new cases in 2017 up from 4% in 2003 according to data from the CDC.^2^ Prostate cancer is typically divided into castrate-sensitive and castrate-resistant subtypes giving distinct challenges in management. Fatalities typically result from metastatic castrate-resistant prostate cancer (mCRPC).

Agents commonly used to manage patients with metastatic prostate cancer include hormonal therapy such as gonadotropin-releasing hormone (GnRH) agonists and antagonists; androgen receptor pathway inhibitors (ARPIs) such as abiraterone, enzalutamide, darolutamide, and apalutamide; chemotherapy such as docetaxel and cabazitaxel; targeted agents such as the poly ADP ribose polymerase (PARP) inhibitors rucaparib and olaparib; immunotherapies such as sipuleucel-T and pembrolizumab; as well as radiopharmaceuticals (which will be the primary focus of this article).

### Radiopharmaceuticals and Differences Between Alpha and Beta Radiation

Radiopharmaceuticals induce cell death by using radiation, typically emitted in the form of alpha or beta particles from radioisotopes, in order to induce single- and double-strand breaks in DNA which initiates apoptosis.^3,4^ Radiopharmaceuticals have their greatest potential benefit in being able to deliver targeted radiation to prostate cancer cells while avoiding normal healthy tissue.

Alpha particles have a short path length of 50-80 μm and high linear energy transfer (LET) of around 100 keV/μm^5^ while beta particles have a long path length of 0.05-12 mm and a much smaller LET of around 0.2 keV/μm.^6^ These differences in path length and energy transfer have the potential to translate into distinct clinical differences. Beta particles have been shown to be less effective than alphas at delivering significant amounts of radiation to smaller tumors^7^ while alpha particles have been shown to be more deleterious. Alphas cause multiple damage sites in DNA, thus having a higher relative biological effectiveness.^8^ Due to the longer path length of beta particles, toxicity to nontarget tissues, namely the bone marrow, is more likely to occur.^9^ Though multiple beta emitters are approved in clinical medicine, only one alpha emitter (radium-223) is approved for clinical use.

### FDA-Approved Therapeutic Radiopharmaceuticals in Prostate Cancer

Multiple radiopharmaceuticals are available for patients with mCRPC. Strontium-89 (89Sr), a beta emitter sold under the trade name Metastron, has been shown to be an effective palliative agent for chemotherapy-refractory patients with bone metastasis.^10^ Samarium-153 (153Sm) lexidronam (EDTMP), a beta emitter sold under the trade name Quadramet, was also shown to provide significant pain relief in patients with bone metastasis.^11^ These two products were FDA approved for palliation in the 1990s but were not shown to have survival benefit.

Radium-223 (223Ra), an alpha emitter sold under the trade name Xofigo, was shown to improve overall survival compared to placebo by 2.8 months (median survival 14.0 months vs. 11.2 months; HR 0.70, 95% CI 0.55-0.88, *P* = .002) in symptomatic bone-predominant mCRPC without visceral metastases.^12^ All main secondary endpoints favored 223Ra over placebo including prolonging time to the first symptomatic skeletal event, time to an increase in the PSA, and improvement in the quality of life based on the FACT-P score. The FDA-approved 223Ra in 2013.

The newest radiopharmaceutical to reach the market was lutetium-177 (177Lu)-PSMA-617 (also known as lutetium Lu-177 vipivotide tetraxetan), a beta emitter sold under the trade name Pluvicto, which was approved by the FDA on March 23, 2022. Patients are eligible for this treatment if they have mCRPC and have previously been treated with an ARPI and with taxane-based chemotherapy and have positive prostate-specific membrane antigen (PSMA) imaging indicating PSMA expression in metastatic lesions.^13^ More details are given on 177Lu-PSMA-617 below.

### PSMA and PSMA-PET Imaging

PSMA is a glutamate carboxypeptidase type II membrane protein produced by the prostate epithelium and specific for and overexpressed in prostate tissue with reduced expression in other organs.^14,15^ Since the discovery of this protein, researchers around the world have investigated targeted diagnostic and therapeutic indications utilizing this protein as a biologic marker.

PSMA-PET imaging is currently being used with two types of radioactive tracers used. 68Ga-labeled (68Ga-PSMA-11) was approved by the FDA on December 1, 2020,^16^ and 18F-labeled (18F-piflufolastat) which was approved by the FDA on May 27, 2021.^17^ Both imaging modalities appear to be equally efficacious with a clinical trial showing noninferiority of 18F to 68Ga in terms of localizing tumors in patients with biochemical recurrence.^18^

### Early Use of PSMA-Based Radioligand Therapy

The first study of PSMA-based radioligand therapy combined radioactive iodine (131I) with the small molecule PSMA binder MIP-1095. A single infusion of 131I-MIP-1095 was given to 28 patients with mCRPC to evaluate for safety and efficacy. PSA levels decreased by>50% in 60.7% of patients, and for patients with bone pain, 84.6% showed a complete or moderated reduction in their symptoms. Based on dosimetry estimates, high uptake was noted in tumor, but significant uptake was also noted in the salivary glands, liver, and kidney. Adverse events (AEs) were low with 25% of patients developing transient xerostomia and there was no effect on renal function noted. Radiation exposure to lymph node and bone metastases were estimated to be upwards of 300 Gy.^19^

## Review of the Key Retrospective Data with 177Lu-PSMA

### German Retrospective Data on 177Lu-PSMA-617

PSMA-617 is a chemically modified DOTA-conjugated PSMA binder associated with high potency binding of PSMA.^20^ Conjugated to 177Lu, 177Lu-PSMA-617 was the first shown to be safe in a small study of 7 patients with mCRPC, with the radiation dose-limiting organ thought to be the parotid glands.^21^

Though the first experience with PSMA-targeted 177Lu dates to 2013 at Bad Burka, this experience was not reported until later. The first report on safety and efficacy came from investigators at University Hospital Bonn and University Hospital Muenster. The authors retrospectively evaluated the results of 10 consecutive patients with mCRPC who were treated with a single dose of 177Lu-DKFZ-617 (later called simply PSMA-617) PSMA between 2013 and 2014. They showed that after 8 weeks, 7 patients (70%) showed a PSA decline with 5 patients (50%) having more than a 50% decline in PSA. Grade 3 or higher hematotoxicity was seen in only one patient and there was no relevant nephrotoxicity or hepatotoxicity.^22^

A subsequent retrospective study, the first evaluating multiple administrations of 177Lu-PSMA-617, was reported by the same group. The authors reviewed 28 consecutive patients who received 50 infusions of 177Lu-PSMA-617 between 2014 and 2015. In their report, a PSA decline was noted in 59% and 75% of patients after 1 and 2 therapies, respectively, and a PSA decline of 50% or greater occurred in 32% and 50%, respectively. The estimated median survival was 29.4 weeks compared to 19.7 weeks in the historical best supportive care group which was statistically significant (HR 44; 95% CI 0.20-0.95; *P* = .031). Authors of the study also noted no significant changes in hematologic or renal parameters.^23^

A larger retrospective multicenter analysis of 145 patients treated with 248 therapy cycles of 177Lu-PSMA-617 at 12 nuclear medicine centers throughout Germany between 2014 and 2015 was conducted. Patients had received 1-4 therapy cycles of treatment. In their analysis, the biochemical response rate (defined as a 50% or greater drop in PSA) was 45% after all therapy cycles. Grade 3 or 4 hematotoxicity occurred in 18 patients (12%) and xerostomia occurred in 11 patients (8%).^24^

## Review of the Key Prospective Data

### Australian Prospective Single-Arm Phase II Data

177Lu-PSMA-617 was first studied in a prospective clinical trial (the LuPSMA trial) at the Peter MacCallum Cancer Centre in Melbourne, Australia. The study was a single-center single-arm phase II clinical trial of men with mCRPC who had progressed on standard-of-care (SOC) treatments including taxane-based chemotherapy and ARPI. Patient selection involved both PSMA PET and fluorodeoxyglucose (FDG) PET. Those patients with lesions detected on FDG PET that were not PSMA avid were excluded from treatment. Patients received up to 4 cycles of 177Lu-PSMA-617 at 6-week intervals. The primary endpoint of the study was PSA response (defined as greater than 50% decline from baseline as per the Prostate Cancer Clinical Trial Work Group criteria) as well as toxicity.^25^

Thirty patients received treatment in this study out of 43 screened patients, thus only 70% of patients were treated. A PSA response was seen in 17 patients (57%). There were no treatment-related deaths in the study and the most common AEs were grade 1 dry mouth in 26 patients (87%), grade 1-2 nausea in 15 patients (50%), and grade 1-2 fatigue in 15 patients (50%).

### VISION Trial

The VISION study was an international prospective open-label phase III study that randomized 831 patients with mCRPC who progressed on 1 or more ARPI and had previously received 1 or 2 lines of taxane chemotherapy, to SOC treatment plus 177Lu-PSMA-617 every 6 weeks for 4-6 cycles, or to treatment with SOC alone.^26^ SOC could include hormonal therapies including ARPIs but no chemotherapy was allowed during the protocol treatment period. Patients were required to have PSMA-PET-positive disease with no PSMA-PET-negative lesions. The definition of a PSMA-PET-negative lesion included several possible criteria. These included a visceral or lytic bone lesion ≥1 cm detected on cross-sectional imaging (CT or MRI) but not seen on PSMA PET. In addition, lymph nodes ≥2.5 cm that were PSMA PET negative were an exclusion criterion. The VISION trial treated approximately 87% of patients that were assessable via PSMA-PET imaging.

Investigators found that patients who received 177Lu-PSMA-617 had a statistically significant improvement in overall survival with a median survival of 15.3 months vs. 11.3 months (HR for death 0.62, 95% CI 0.52-0.74, *P* < .001). Radiographic progression-free survival (rPFS) also improved and favored 177Lu-PSMA-617. Key secondary endpoints including time to the first symptomatic skeletal event or death, complete responses based on RECIST 1.1, confirmed decreases in PSA, time to deterioration in the FACT-P total score, and BPI-SF pain intensity score, all favored 177Lu-PSMA-617.

Toxicities of grade 3 or higher were experienced by 52.7% of patients compared to 38.0% of patients in the SOC arm. The most common all-grade toxicities with 177Lu-PSMA-617 were fatigue (43.1%), dry mouth (38.8%-0% grade 3 or higher), and nausea (35.3%). The most common grade 3 or higher toxicities included anemia (12.9%), thrombocytopenia (7.9%), and leucopenia (7.8%).

The positive results of this study lead to the FDA approval of Pluvicto (lutetium Lu-177 vipivotide tetraxetan, also known as 177Lu-PSMA-617) on March 23, 2022. The role of SOC is not clear but those with an ARAT + the isotope tended to fare better in the survival analysis, and the importance of concomitant ARATs still needs to be determined.

### TheraP Trial

The TheraP study was an open-label phase II clinical trial at 11 centers in Australia which randomized 200 patients with mCRPC for whom cabazitaxel was considered the next most appropriate treatment (progression on docetaxel was required) to receive cabazitaxel intravenously every 3 weeks (up to 10 cycles) or to receive 177Lu-PSMA-617 every 6 weeks (up to 6 cycles). Inclusion in this study required a positive 68Ga-PSMA PET and patients were required to also have an 18F-FDG PET with no sites of disease that were FDG-avid but PSMA negative.^27^

Of the patients with PET screening in TheraP, approximately 27% were excluded from treatment. Investigators found that patients who received 177Lu-PSMA-617 were more likely to receive a PSA response (66% vs. 37% in the intent-to-treat group, *P* < .0001). Updated survival analysis was presented at a 2022 meeting, and overall survival was similar between 177Lu-PSMA-617 and cabazitaxel at 19.1 months versus 19.6 months was not significantly different between the 2 arms.^28^ The finding of survival equivalence in a PSMA-selected patient population was surprising to some but it is well known that cabazitaxel is an important life-prolonging therapy in mCRPC.

Grade 3-4 AEs occurred in 32 (33%) of men who received 177Lu-PSMA-617 versus 45 (53%) of men in the cabazitaxel group. All-grade toxicities that were more common in the 177Lu-PSMA-617 were dry mouth (60% vs. 21%), dry eyes (30% vs. 4%), and thrombocytopenia (18% vs. 5%). All-grade toxicities that were more common with cabazitaxel were diarrhea (52% vs. 18%), neuropathy (26% vs. 10%), and dysgeusia (27% vs. 12%).

## Trials in Progress with 177Lu-PSMA Radiopharmaceuticals

### PSMAfore

PSMAfore (NCT04689828) is an open-label, randomized phase III clinical trial that will assess the effectiveness of 177Lu-PSMA-617 in patients with mCRPC in the pre-taxane setting. PSMA PET selection will be similar to that done in VISION. Approximately 450 participants will be randomized to receive 177Lu-PSMA-617 or to an ARPI. All patients are required to have progressed on only 1 ARPI (abiraterone, enzalutamide, darolutamide, or apalutamide). The primary outcome of the study is rPFS.^29^ A crossover is available after progression in the control group.

Novartis has announced via a press release that PSMAfore has completed accrual and has shown statistically significant improvement in rPFS with findings to be presented in 2023.

### PSMAddition

PSMAddition (NCT04720157) is an open-label, randomized phase III clinical trial that will determine the efficacy of 177Lu-PSMA-617 in combination with SOC versus SOC alone with patients with mCSPC. SOC in this case will be ADT + an ARPI. Approximately 1126 participants will be randomized to 177Lu-PSMA-617 + ARPI + ADT or to ARPI + ADT. Patients are required to have a metastatic disease not previously treated with hormonal therapy and those patients with rapidly progressive disease in need of taxane-based chemotherapy will be excluded. The primary outcome of the study is rPFS.^30^ A crossover is allowed for those who progress on SOC alone.

### ECLIPSE

ECLIPSE (NCT05204927) is an open-label, randomized phase III clinical trial designed to determine the efficacy of 177Lu-PSMA-I&T (produced by Curium US LLC) compared to SOC ARPI in men with mCRPC. Patients must have received only 1 line of ARPI therapy and have progressed on it. Patients are excluded if they have previously received chemotherapy or if they have a homologous recombination repair gene mutation and have not yet received olaparib or rucaparib. Approximately 400 men with mCRPC will be randomized to receive Lu-177-PSMA-I&T or abiraterone or enzalutamide. The primary outcome of the study is rPFS.^31^

### SPLASH

SPLASH (NCT04647526) is an open-label, randomized phase III clinical trial that will determine the effectiveness of [Lu-177]-PNT2002 (also known as 177Lu-PSMA-I&T produced by POINT Biopharma) versus abiraterone or enzalutamide in patients with mCRPC. Patients are required to have been treated and progressed on 1 ARPI and prior chemotherapy for castrate-resistant disease is exclusionary. The study will begin with a 25-patient safety and dosimetry lead followed by randomization of approximately 390 patients to either [Lu-177]-PNT2002 or enzalutamide or abiraterone. [Lu-177]-PNT2002 will be given every 8 weeks for a maximum of 4 cycles. The primary outcome of the study is rPFS.^32^

### BULLSEYE

177Lu-PSMA-I&T was also planned to be studied in the BULLSEYE phase II clinical trial (NCT04443062) as metastasis-directed therapy in patients with prostate cancer and oligometastatic castrate-sensitive disease (defined as 5 or less metastasis on PSMA-PET) as a randomized controlled trial where patients receive the interventional arm of receiving 2 cycles of 177Lu-PSMA-I&T versus the SOC.^33^ However, due to COVID-19-related issues (specifically personnel shortage, increased remote working, and decreased hospital resources), the production of 177Lu-PSMA-I&T was hindered and a protocol amendment replacing 177Lu-PSMA-I&T for 177Lu-PSMA-617 was made. The primary outcome measure of this study is disease progression defined by 100% PSA increase or clinical progression.^34^

The ongoing phase III clinical trials utilizing 177Lu-PSMA-617 in mCRPC are shown in Table 1.

**Table 1.** Ongoing phase III clinical trials utilizing 177Lu-PSMA-617 in mCRPC

Study name	Disease setting	NCT number	Trial locations	Comparitor arm	Primary outcome measure
An International Prospective Open-label, Randomized, Phase III Study Comparing 177Lu-PSMA-617 in Combination With SoC, Versus SoC Alone, in Adult Male Patients With mHSPC (PSMAddition)	Metastatic hormone naive prostate cancer	NCT04720157	International	ARPI + ADT	rPFS
177Lu-PSMA-617 vs. Androgen Receptor-directed Therapy in the Treatment of Progressive Metastatic Castrate Resistant Prostate Cancer (PSMAfore)	Metastatic castration-resistant prostate cancer	NCT04689828	International	ARPI + ADT	rPFS
Study Evaluating mCRPC Treatment Using PSMA [Lu-177]-PNT2002 Therapy After Second-line Hormonal Treatment (SPLASH)	Metastatic castration-resistant prostate cancer	NCT04647526	International	Abiraterone or Enzalutamide + ADT	rPFS
Lu-177-PSMA-I&T for Metastatic Castration-Resistant Prostate Cancer	Metastatic castration-resistant prostate cancer	NCT05204927	United States	Abiraterone or Enzalutamide + ADT	rPFS
Lu-177-PSMA-I&T for Metastatic Castration-Resistant Prostate Cancer	Metastatic castration-resistant prostate cancer	NCT05204927	United States	Abiraterone or Enzalutamide + ADT	rPFS

Abbreviation: rPFS, radiographic progression-free survival.

## Combination Therapies with 177Lu-PSMA-617

### Radiation

The PROQURE-1 study (NCT05162573) is being conducted to evaluate the effectiveness of 177Lu-PSMA-617 in combination with external beam radiation therapy (EBRT) in patients with node-positive (N1M0) prostate cancer for which SOC is typically EBRT with 2-3 years of ADT. This will be a multicenter prospective phase I dose escalation study with 3 dose levels where 177Lu-PSMA-617 is given concurrently with EBRT. Enrollment will consist of 18 patients in the Netherlands. The primary outcome measure is the maximum tolerated dose (MTD) of 177Lu-PSMA-617 when given concurrently with EBRT.^35^

### Immunotherapy

177Lu-PSMA-617 has been studied in combination with pembrolizumab in patients with mCRPC who are chemotherapy naïve post at least 1 ARPI (NCT03805594). In that single-arm phase Ib clinical trial of 18 patients, objective response was seen in 8 patients (44%) with median rPFS of 6.5 months. A PSA decline of 50% or more from baseline was seen in 28% of patients. There were no dose-limiting toxicities (DLTs) and only 1 grade 3 or higher AE was noted which was inflammatory arthritis.^36^

Two other studies are looking at combining immunotherapy with 177Lu-PSMA. The PRINCE study (NCT03658447) is a single-arm phase Ib/II clinical trial evaluating the combination of pembrolizumab and 177Lu-PSMA. Estimated enrollment will be 37 patients where the primary outcome measured is PSA response, safety, and tolerability.^37^ The other study is the EVOLUTION study (NCT05150236) which will be a phase II randomized trial of 177Lu-PSMA-617 versus 177Lu-PSMA-617 with ipilimumab and nivolumab for patients with mCRPC. Estimated enrollment will be 110 patients and the primary outcome measure is PSA PFS.^38^

### Enzalutamide

The ENZA-p study (NCT04419402) is a randomized multicenter phase II trial that will enroll men with mCRPC to treatment with enzalutamide 160 mg daily versus enzalutamide 160 mg daily plus 177Lu-PSMA-617 on days 15 and 57 with 2 subsequent doses of if 68Ga-PSMA PET is still positive on day 92. Estimated enrollment will be 160 patients and the primary endpoint of this study is PSA PFS.^39^

### Docetaxel

The UpFrontPSMA study (NCT04343885) is another ongoing study looking at the sequential use of 177Lu-PSMA-617 every 6 weeks for 2 cycles followed by docetaxel 75 mg/m^2^ q3w × 6 cycles compared to SOC (docetaxel alone) in patients with newly diagnosed high-volume mCSPC. The clinical trial will recruit an estimated 140 patients at 11 sites in Australia. The primary outcome measure of this study will be an undetectable PSA rate at 12 months.^40^

### Cabazitaxel

The LuCAB study (NCT05340374) is a single-arm single-center open-label phase I/II clinical trial designed to assess the safety and efficacy of cabazitaxel in combination with 177Lu-PSMA-617 in patients with mCRPC. Estimated enrollment will be 44 patients in Australia where the primary outcome measures will be the number of patients with DLTs, the MTD, and the recommended phase II dose (RP2D).^41^

### Olaparib

The LuPARP study (NCT03874884) is a phase I dose escalation and dose expansion study evaluating the combination of Olaparib with 177Lu-PSMA-617. An estimated 52 patients will be enrolled in Australia. The primary outcome measures of this study are the DLTs, MTD, and R2PD of the combination.^42^

### Radium-223

The AlphaBet study (NCT05383079) is a single-arm open-label phase I/II study being conducted to evaluate the combination of Radium-223 and 177Lu-PSMA-I&T in patients with mCRPC. Estimated enrollment is 36 patients in Australia. The primary outcome measures are DLTs, MTD, RP2D, and PSA response.^43^

### Abemaciclib

The UPLIFT study (NCT05113537) is a phase I/II study being conducted to determine if abemaciclib (a CDK4/CDK6 inhibitor), given on days 1-14 as a pretreatment to 177Lu-PSMA which is given on day 15, may increase the efficacy of 177Lu-PSMA-617. Estimated enrollment is 30 patients. The primary outcome measures are DLT, RP2D, and the change in maximum SUV on 68Ga-PSMA-11 PET.^44^

The clinical trials using combination therapies with 177Lu-PSMA-617 can be found in Table 2.

**Table 2.** Combination studies utilizing 177Lu-PSMA-617,

Study name	Disease setting	NCT number	Clinical trial phase	Location	Primary outcome measure
EBRT + Lu-PSMA for N1M0 Prostate Cancer (PROQURE-1)	N1M0	NCT05162573	1	Netherlands	MTD
177Lu-PSMA-617 Therapy and Olaparib in Patients With Metastatic Castration Resistant Prostate Cancer (LuPARP)	Metastatic castration-resistant Prostate Cancer	NCT03874884	1	Australia	DLT MTD RP2D
177Lu-PSMA-617 and Pembrolizumab in Treating Patients With Metastatic Castration-Resistant Prostate Cancer	Metastatic castration-resistant prostate cancer	NCT03805594	1	United States	RP2D ORR
Abemaciclib Before 177Lu-PSMA-617 for the Treatment of Metastatic Castrate-Resistant Prostate Cancer (UPLIFT)	Metastatic castration-resistant prostate cancer	NCT05113537	1/2	United States	DLT MTD RP2D Change in maximum standardized uptake value (SUVmax)
Combination of Radium-223 and Lutetium-177 PSMA-I&T in Men With Metastatic Castration-Resistant Prostate Cancer (AlphaBet)	Metastatic castration-resistant prostate cancer	NCT05383079	1/2	Australia	DLT MTD RP2D PSA response rate
Cabazitaxel in Combination With 177Lu-PSMA-617 in Metastatic Castration-resistant Prostate Cancer (LuCAB)	Metastatic castration-resistant prostate cancer	NCT05340374	1/2	Australia	DLT MTD RP2D
PRINCE (PSMA-lutetium Radionuclide Therapy and ImmuNotherapy in Prostate CancEr) (PRINCE)	Metastatic castration-resistant prostate cancer	NCT03658447	1/2	Australia	PSA response rate Safety/Tolerability
Enzalutamide With Lu PSMA-617 Versus Enzalutamide Alone in Men With Metastatic Castration-resistant Prostate Cancer (ENZA-p)	Metastatic castration-resistant prostate cancer	NCT04419402	2	Australia	PSA Progression-Free Survival
EVOLUTION: 177Lu-PSMA Therapy Versus 177Lu-PSMA in Combination With Ipilimumab and Nivolumab for Men With mCRPC (ANZUP2001)	Metastatic castration-resistant prostate cancer	NCT05150236	2	Australia	PSA progression free survival
In Men With Metastatic Prostate Cancer, What is the Safety and Benefit of Lutetium-177 PSMA Radionuclide Treatment in Addition to Chemotherapy (UpFrontPSMA)	Metastatic hormone naive prostate cancer	NCT04343885	2	Australia	Undetectable PSA rate at 12 months

Abbreviations: DLT, dose-limiting toxicity; MTD, maximum tolerated dose; ORR, objective response rate; RP2D, recommended phase II dose.

## New Agents and Isotopes: Radiolabeled Monoclonal Antibodies

### 225Ac-J591

225Ac is an alpha emitter and is being studied conjugated to J591 which is a monoclonal antibody to the extracellular domain of PSMA.^45^ 225Ac-J591 was studied in a phase I clinical trial (NCT03276572) of patients with progressive mCRPC patients who were treated with a single infusion of 225Ac-J591 (13.3 KBq/kg with a planned escalation up to 93.3 KBq/kg). Patients with any PSA decline was noted to be 22 (68.7%) and the patients with more than 50% PSA decline was 12 (37.5%). One patient was noted to have a DLT (grade 4 anemia and thrombocytopenia). In addition, 4 patients (12.5%) developed grade 3 thrombocytopenia and 2 (6.2%) developed grade 3 neutropenia. All high-grade AEs were hematologic. In patients with patient-reported outcomes (PRO) data, pain scores improved by 12 weeks.^46^

225Ac-J591 is being further studied in a phase I clinical trial evaluating the safety of a single fractionated dose as well a single dose every 6 weeks for up to 4 treatments (NCT04506567). There is an estimated enrollment of 105 patients and the primary outcomes will be DLT, MTD, and R2PD.^47^ Another study is looking at the combination of 225Ac-J591 with pembrolizumab and with an ARPI in a phase I/II clinical trial (NCT04946370). There is an estimated enrollment of 76 patients where the primary outcome measures are DLT, RP2D, and efficacy.^48^

### 177Lu-J591

177Lu has also been studied conjugated with J591. In a phase Ib/II study (NCT00538668), 49 patients received fractionated doses of 177Lu-J591 (ranging from 20 to 45 mCi/m^2^) for 2 infusions 2 weeks apart.^49^ There was no selection for PSMA expression before enrollment in this study. At the highest dose of 45 mCi/m^2^, 87.5% of patients achieved a PSA decrease with 29.4% of patients with a PSA decrease of >50%, and median survival in this group was 42.3 months. At this dose, 35.3% of patients experienced grade 4 neutropenia and 58.8% of patients experienced thrombocytopenia. Considering all 49 patients, any PSA decline was noted in 55.1%, a greater than 50% decline in PSA was noted in 16.3% of patients, and median overall survival was 22.9 months.^50^

### TLX591

TLX591 is a radioimmunoconjugate composed of a monoclonal antibody to PSMA, rosopatamab, linked to 177Lu via a chelating agent DOTA. Also known as 177Lu-DOTA-HuJ591-CHO, TLX591 is currently being investigated for its safety, biodistribution, and dosimetry properties in the ProstACT Select study, a phase I trial with an estimated enrollment of 50 patients (NCT04786847).^51^ A phase III trial is also planned to compare the use of TLX591 as 2 single intravenous injections of 76 mCi 14 days apart plus SOC versus SOC alone for patients with PSMA expressing mCSPC in the PROSTACT trial (NCT04876651). This study has an estimated enrollment of 387 patients with the primary outcome being rPFS.^52^

### 225Ac-TLX592

TLX592 is another PSMA-targeted monoclonal antibody, HuX592r, radiolabeled with 64Cu via the chelating agent DOTA. Also known as 64Cu-DOTA-TLX592, it is currently being studied in the CUPID study (NCT04726033) which is a phase I clinical trial evaluating the safety, pharmacokinetics, biodistribution, and dosimetry with an estimated enrollment of 15 patients.^53^ Following CUPID, plans are to explore TLX592 conjugated to 225Ac.

### 227Th-PSMA-TTC

227Th-PSMA-TTC represents a targeted alpha radiation therapy, with the alpha particle emitter thorium-227 (half-life 18.7 days) linked to a fully humanized monoclonal antibody to PSMA, PSMA-TTC (BAY 2315497). In preclinical studies, 227-Th-PSMA-TTC was shown to inhibit cancer growth through the induction of double-stranded DNA breaks.^54^

The clinical trials employing radiolabeled monoclonal antibodies are seen in Table 3.

**Table 3.** Novel radiolabeled monoclonal antibodies.

Agent	Type of radiation	NCT number	Clinical trial phase	Location	Primary outcome measure
225Ac-J591	Alpha	NCT03276572	1	United States	MTD DLT
		NCT04506567	1/2	United States	DLT MTD RP2D
227Th-PSMA-TTC	Alpha	NCT03724747	1	International	MTD
177Lu-J591	Beta	NCT00538668	1	United States	PK Dosimetry Preliminary efficacy Myelotoxicity
TLX591	Beta	NCT04786847	1	Australia	Treatment-Related Adverse Events
		NCT04876651	3	Australia	rPFS
TLX592	Beta	NCT04726033	1	Australia	Treatment-Related Adverse Events PK/PD Dosimetry

Abbreviations: DLT, dose-limiting toxicity; MTD,maximum tolerated dose; RP2D, recommended phase II dose; rPFS, radiographic progression-free survival.

## New Agents and Isotopes: Radiolabeled Small-Molecule Binders

### I-131-1095

The ARROW trial (NCT03939689) is an open-label randomized phase II study evaluating the efficacy of enzalutamide in combination with I-131-1095 compared to enzalutamide alone, in patients with progressive mCRPC who have previously received abiraterone. I-131 is radioactive iodine commonly used to treat patients with thyroid disease and is a beta emitter.^55^ 1095 is a PSMA binder that binds with high affinity and is internalized by the cell. Estimated enrollment is 120 patients. The primary objective of this study is efficacy defined by PSA.^56^

### 177Lu-DOTA-N3-CTT1403

CTT1403 is a PSMA-targeted 177Lu-labeled radiotherapy being studied in a phase I clinical trial in men with PSMA avid mCRPC post at least 1 ARPI (NCT03822871). Estimated enrollment will be 40 patients. The primary outcome of the study will be DLTs.^57^

### 225Ac-PSMA-617

The AcTION study (NCT04597411) is a phase I study of 225Ac-PSMA-617, where the alpha emitter 225Ac is conjugated to PSMA binder PSMA-617, in patients with mCRPC who either have or have not previously received 177Lu-PSMA-617 radioligand therapy or 177Lu-PSMA-I&T. Estimated enrollment will be 60 patients and the primary outcome measure of this study is the RP2D.^58^ The trial is being conducted only in Australia and South Africa.

### 177Lu-PSMA-R2

The PROter study (NCT03490838) is a phase I/II dose escalation study of 177Lu-PSMA-R2 in patients with mCRPC designed to evaluate the safety, tolerability, and efficacy of this compound. As of July 6, 2022, the study was halted in phase I by the sponsor (Advanced Accelerator Applications) and phase II was not initiated.^59^

### 67Cu-PSMA

The SECuRE study (NCT04868604) is a single-arm phase I/II clinical trial evaluating the safety and efficacy of 67Cu-SAR-bisPSMA in patients with mCRPC. The 67Cu is a radioisotope of copper that emits beta particles.^60,61^ This study does require patients to have positive PSMA expression defined by a positive 64Cu-SAR-bisPSMA PET/CT scan. An estimated 44 patients will be enrolled with the primary outcomes evaluating safety, tolerability, and efficacy.^62^

The human studies incorporating radiolabeled novel small-molecule binders are displayed in Table 4.

**Table 4.** Novel radiolabeled small-molecule binders.

Agent	Type of radiation	NCT number	Clinical trial phase	Location	Primary outcome measure
225Ac-PSMA-617	Alpha	NCT04597411	1	International	R2PD
I-131-1095	Beta	NCT03939689	2	International	PSA response
177Lu-DOTA-N3-CTT1403	Beta	NCT03822871	1	United States	DLT
177Lu-PSMA-R2	Beta	NCT03490838	1	International	DLT PSA response
PSMA Cu-64/67 trial	Beta	NCT04868604	1/2	United States	Safety Tolerability Efficacy

Abbreviations: DLT, dose-limiting toxicity; RP2D, recommended phase II dose.

## Conclusion

Radiopharmaceuticals have been used in prostate cancer for decades with known efficacy in palliation of bone metastasis. The FDA approval of the most recent radiopharmaceutical, 177Lu-PSMA-617, shows a clear overall survival benefit compared to the best SOC. Current studies are ongoing to evaluate the effectiveness of 177Lu-PSMA-617 in earlier lines of therapy, such as after only 1 line of therapy with an ARPI as in the PSMAfore study, or in mCSPC in combination with ADT and an ARPI as in the PSMAddition study. Studies are also ongoing with 177Lu-PSMA-617 in combination with other treatments which may produce a synergistic effect. As described, there are many clinical trials ongoing that are modifying the 177Lu-PSMA-617 to possibly achieve improved results. Studies are also ongoing looking at swapping the 177Lu for another radioactive agent, such as 225Ac (an alpha emitter) which may have benefits over the beta-emitting 177Lu. Other studies are looking at J591, a monoclonal antibody to PSMA which has unique differences compared to small-molecule PSMA binders. Overall, PSMA-based radioligand therapy has been shown to be an effective and life-prolonging therapy and should be considered a treatment option for selected men with mCRPC. As described, there are many ongoing studies further evaluating PSMA radioligand therapy in Australia, South Africa, Europe, and the United States, representing a truly global effort to improve prostate cancer care.

## Data Availability

No new data were generated or analyzed in support of this research.
